# A comparative analysis of growth and developmental outcomes in infants with proliferative hemangiomas treated with oral propranolol versus prednisone

**DOI:** 10.3389/fpubh.2026.1915868

**Published:** 2026-09-11

**Authors:** Yanli Niu, Dan Song, Jing Li, Liang Wang, Lei Guo

**Affiliations:** Department of Vascular Anomalies and Interventional Radiology, Ji’nan Children’s Hospital (Children’s Hospital Affiliated to Shandong University), Jinan, Shandong, China

**Keywords:** growth and development, infant health, infantile hemangioma, pediatrics, prednisone, propranolol

## Abstract

**Objective:**

To investigate the clinical efficacy, adverse reactions, and physical growth and neurodevelopment effects of oral propranolol in infants with proliferative hemangiomas and to compare these outcomes with those of oral prednisone.

**Methods:**

A retrospective analysis was conducted on 150 infants with proliferative infantile hemangiomas (IHs) treated at our hospital between October 2023 and October 2025. On the basis of outpatient treatment regimens, 77 infants who received oral propranolol were included in the study group, and 73 infants who received oral prednisone were included in the control group. Clinical data, treatment outcomes, adverse reactions, height, weight, head circumference, and neurodevelopment (assessed by using the Gesell Developmental Scale) were collected from medical records. After 3–6 months of treatment, clinical efficacy was evaluated with the Achauer efficacy grading system, and adverse reactions, physical growth parameters, and neurodevelopmental indicators were compared between the two groups.

**Results:**

After 3–6 months of treatment, the clinical efficacy in the study group was significantly better than that in the control group (*p* < 0.05). The incidence of common adverse reactions, including hypotension, hypoglycemia, constipation, diarrhea, and vomiting, did not significantly differ between the two groups (*p* > 0.05). However, the incidence of respiratory tract infections was significantly lower in the study group than in the control group (*p* < 0.05). The infants in the study group also had significantly greater height, weight, and head circumference than those in the control group (*p* < 0.05). Neurodevelopmental assessment showed that developmental quotients in gross motor, fine motor, adaptive behavior, language, and personal–social domains were all significantly higher in the study group than in the control group (*p* < 0.05).

**Conclusion:**

Compared with oral prednisone, oral propranolol demonstrates superior therapeutic efficacy in the treatment of proliferative IHs and is associated with fewer respiratory infections and better physical and short-term neurodevelopmental outcomes. These findings suggest that propranolol may offer advantages over prednisone in terms of efficacy, safety, and developmental outcomes, supporting its use as a preferred first-line therapy for proliferative IHs. However, given the limited sample size and short follow-up of this study, large prospective studies are warranted to confirm these observations.

## Introduction

Infantile hemangioma (IH) is the most common benign vascular tumor in children, with an incidence of approximately 4–10% (1, 2). This condition occurs more frequently in females than in males and typically undergoes rapid proliferation within weeks to months after birth, potentially involving the skin, subcutaneous tissue, and even vital organs (1, 3). The clinical manifestations of hemangiomas are diverse, ranging from superficial localized lesions to deep or mixed types. Some infants may experience complications, such as bleeding, ulceration, functional impairment, or cosmetic deformity, and severe cases may even become life-threatening (4, 5).

Before the introduction of propranolol, systemic therapy for proliferative hemangiomas relied primarily on corticosteroids, such as oral prednisone (6), which were considered the first-line pharmacological option at that time. Corticosteroids exert their effects by reducing inflammation, inhibiting endothelial cell proliferation, and promoting vascular regression (7). However, their long-term use, typically at doses of 2–3 mg/kg/day for more than 4–8 weeks, may lead to adverse effects, such as impaired growth and development, immunosuppression, elevated blood glucose levels, and osteoporosis, limiting their clinical application (8, 9). These side effects are generally dose and duration dependent, with cumulative exposure and high doses increasing the risk of systemic complications.

Since Léauté-Labrèze et al. reported the efficacy of oral propranolol for treating infantile hemangiomas (IHs) in 2008, propranolol has rapidly become the first-line therapy for proliferative IHs because of its rapid onset, reliable efficacy, and high safety profile (10). Propranolol primarily acts through blocking β-adrenergic receptors, inducing vasoconstriction, inhibiting endothelial cell proliferation, promoting apoptosis, and reducing angiogenic factor expression, thereby shrinking lesions and improving their color and texture (11). In addition, propranolol may further promote hemangioma regression by modulating local inflammation and the vascular microenvironment (12).

Although numerous studies have confirmed the advantages of propranolol in reducing hemangioma size, improving appearance, and lowering recurrence rates, its effects on infant growth and neurodevelopment remain controversial (13). Some studies suggest that its long-term or high-dose use may potentially affect physical growth, head circumference increase, and neuromotor development; however, systematic investigations on this issue remain limited (14).

Therefore, in this study, we aim to analyze retrospectively infants with proliferative IHs who received oral propranolol or oral prednisone in our outpatient clinic, comparing the two groups in terms of clinical efficacy, adverse reactions, physical growth, and neurodevelopment. Our objective is to evaluate the overall efficacy and safety of propranolol. Our results are intended to provide evidence-based guidance for clinical treatment selection and offer a reference for the safe use of propranolol in infancy.

## Materials and methods

### Study subjects

This study included 150 infants with proliferative IHs treated at our hospital from October 2023 to October 2025. In accordance with outpatient treatment regimens, 77 infants who received oral propranolol were assigned to the study group, and 73 infants who received oral prednisone were assigned to the control group. This retrospective study was conducted in accordance with the principles of the Declaration of Helsinki. This work was approved by the Ethics Committee of Ji’nan Children’s Hospital (Approval Number: SDFE-IRB/P-2023060). The requirement for informed consent was waived by the ethics committee because this study involved the retrospective analysis of anonymized clinical data and posed no additional risks to participants. The baseline characteristics of the two groups are summarized in Table 1.

**Table 1:** Comparison of baseline characteristics.

Factor	Control group (*n* = 73)	Study group (*n* = 77)	*t*/χ^2^	*p*
Gender				
Male	28	27	0.175	0.676
Female	45	50		
Months of age	2.75 ± 0.43	2.81 ± 0.50	0.822	0.412
Body length (cm)	58.80 ± 2.55	59.45 ± 2.70	1.521	0.130
Body weight (kg)	5.71 ± 0.68	5.65 ± 0.70	0.517	0.606
Head circumference (cm)	38.59 ± 2.55	39.00 ± 3.28	0.849	0.397
Location				
Trunk	29	32	0.328	0.849
Limbs	22	25		
Head and neck	22	20		
Hemangioma type				
Single hemangioma	61	67	14.770	0.002
Multiple hemangiomas	12	10		
Involvement of the subcutaneous fat layer				
Yes	56	61	0.137	0.711
No	17	16		
Premature infant				
Yes	16	14	0.327	0.567
No	57	63		

#### Inclusion criteria

Outpatient treatment during the study period: visited our hospital outpatient clinic between October 2023 and October 2025, were diagnosed with proliferative IH, and received systemic drug therapy.

Confirmed diagnosis: with clinical manifestations consistent with IH and confirmed to be in the proliferative phase by a dermatologist, pediatric specialist, or ultrasound examination.

Clear treatment regimen: with complete records showing that the infants received either oral propranolol or oral prednisone, with documented drug doses and treatment duration sufficient for efficacy and growth analysis.

Complete medical records: with medical records containing general information, treatment details, and follow-up information (e.g., weight, height, and head circumference) sufficient for analysis.

Informed consent was obtained from parents or legal guardians.

#### Exclusion criteria

Incomplete medical records: with missing key clinical information that prevents analysis.

Nonconforming treatment regimen: with the concurrent use of other systemic therapies that may affect hemangioma progression or growth evaluation (e.g., other hormones, β-blockers, and antiangiogenic drugs) with unclear impact.

Severe underlying diseases: With significant cardiovascular or pulmonary disease and metabolic or endocrine disorders (e.g., diabetes and thyroid or adrenal disorders) that may affect growth or the safety of propranolol/prednisone use.

Complex vascular malformations or syndromes: with conditions like PHACE syndrome or Kasabach–Merritt phenomenon, whose condition or treatment may influence growth and development assessment.

With known intolerance or severe adverse reactions to propranolol or corticosteroids. Infants with a history of anemia, chronic diseases (e.g., congenital heart disease, chronic lung disease, and metabolic disorders), or conditions that could independently affect growth or neurodevelopment were excluded on the basis of medical record review.

Other conditions deemed unsuitable for analysis: with any other factors that, in the judgment of the investigators, could markedly affect the accuracy of study results.

### Treatment methods

This study is a retrospective analysis. In accordance with the actual systemic treatment received by infants in our outpatient clinic, cases were divided into a study group receiving oral propranolol and a control group receiving oral prednisone. All treatment regimens were determined by the attending physicians at the time on the basis of the infants’ condition and parental preferences.

#### Study group: oral propranolol treatment

Infants in the study group received oral propranolol, with a conventional initial dose of 1.0 mg/kg/day divided into 2–3 daily administrations. The dose was gradually increased to 2.0–3.0 mg/kg/day in accordance with tolerance and lesion response. During treatment, the clinical monitoring of heart rate, blood pressure, blood glucose, and potential adverse reactions was recorded in accordance with clinical guidelines. The usual treatment duration was 3–6 months, with some cases extended on the basis of lesion response, as documented in medical records.

#### Control group: oral prednisone treatment

Infants in the control group received oral prednisone at a typical dose of 1.0–2.0 mg/kg/day, administered once daily or as divided doses. Once the infants’ condition stabilized, the attending physician gradually tapered the dose in accordance with lesion control and adverse reactions. The conventional treatment course was approximately 4–8 weeks, followed by gradual dose reduction until discontinuation as directed by the physician.

### Routine follow-up and monitoring

All infants were monitored during outpatient follow-up for growth parameters (weight, height, and head circumference), changes in lesion volume, and drug-related adverse reactions. Follow-up frequency was determined by the attending physician on the basis of clinical condition, typically every 2–4 weeks. Electrocardiogram monitoring, blood glucose measurements, and adverse event records were included as part of safety assessment if available. Generally, follow-up visits were scheduled weekly in the first month, biweekly in the second month, and monthly after 3 months, with adjustments made in accordance with individual patient needs. The treatment duration for both groups was 3–6 months.

### Outcome measures

#### Clinical efficacy evaluation

After 3–6 months of treatment, the clinical efficacy of IH treatment in both groups was assessed by using the Achauer efficacy grading system. This method evaluates treatment outcomes on the basis of lesion volume, color, and appearance and categorizes efficacy into four grades, namely, Grade I (poor, <25% improvement), Grade II (moderate, 25–50% improvement), Grade III (good, 51–75% improvement), and Grade IV (excellent, >75% improvement), with high grades indicating good therapeutic effects.

#### Adverse reaction monitoring

Medical records from follow-up visits were reviewed to compare the incidence of adverse reactions during treatment between the two groups. The main adverse reactions included hypotension, hypoglycemia, constipation, diarrhea, vomiting, and respiratory tract infections. The number of cases for each adverse reaction was recorded, and differences in the overall incidence of complications between groups were analyzed.

#### Growth indicators

After 3–6 months of treatment, growth parameters, including height (cm), weight (kg), and head circumference (cm), were compared between the two groups. These indicators were extracted from follow-up records or growth monitoring data.

#### Neurodevelopmental indicators

Neurodevelopmental status after 3–6 months of treatment was assessed with the Gesell Developmental Schedules, a standardized tool that is widely used in Chinese pediatric populations for assessing early childhood development. The evaluated functional domains included gross motor, fine motor, adaptive behavior, language, and personal–social behavior. Developmental quotient (DQ) scores for each domain were recorded and compared between the two groups. The Gesell Scale has demonstrated good reliability and validity in the Chinese infant population.

### Statistical analysis

Statistical analyses were performed by using SPSS Version 27.0. Independent t-tests and χ^2^ tests were applied as appropriate. Continuous variables were tested for normality and are presented as mean ± standard deviation (x̄ ± s). Categorical data are presented as frequencies and percentages (n/%). *p* < 0.05 was considered statistically significant.

## Results

### Comparison of baseline characteristics between the two groups

The control group included 28 male and 45 female infants, with a mean age of 2.75 ± 0.43 months, mean height of 58.80 ± 2.55 cm, mean weight of 5.71 ± 0.68 kg, and mean head circumference of 38.59 ± 2.55 cm. Lesion distribution included 29 cases on the trunk, 22 on the limbs, and 22 on the head and neck. A total of 61 infants had a single hemangioma, 12 infants had multiple hemangiomas, 56 cases involved the subcutaneous fat layer, and 16 infants were preterm.

The study group included 27 male and 50 female infants, with a mean age of 2.81 ± 0.50 months, mean height of 59.45 ± 2.70 cm, mean weight of 5.65 ± 0.70 kg, and mean head circumference of 39.00 ± 3.28 cm. Lesion distribution included 32 cases with lesions on the trunk, 25 on the limbs, and 20 on the head and neck. A total of 67 infants had a single hemangioma, 10 infants had multiple hemangiomas, 61 cases involved the subcutaneous fat layer, and 14 infants were preterm. No statistically significant differences were found between the two groups (*p* > 0.05) (Table 1).

### Comparison of treatment outcomes after 3–6 months

After 3–6 months of treatment, the therapeutic effects of the two drugs were evaluated in both groups of infants by using the Achauer efficacy grading system. The results showed that the treatment effect in the study group was significantly better than that in the control group (*p* < 0.05) (Table 2).

**Table 2:** Comparison of Achauer efficacy grading between the two groups after 3–6 months of treatment.

Factor	Control group (*n* = 73)	Study group (*n* = 77)	*t*/χ^2^	*p*
Grade I	11	4	14.770	0.002
Grade II	20	7		
Grade III	22	34		
Grade IV	20	32		

### Comparison of adverse reactions between the two groups

The occurrence of adverse reactions, including respiratory changes, hypotension, hypoglycemia, constipation, diarrhea, and vomiting, was observed in both groups during follow-up. Of the infants in the control group, one experienced hypotension, five had hypoglycemia, one had constipation, eight had diarrhea, one had vomiting, and 16 had respiratory tract infections. Of the infants in the study group, 0 experienced hypotension, 0 had hypoglycemia, 0 had constipation, three had diarrhea, one had vomiting, and seven had respiratory tract infections. The incidence of common adverse reactions (hypotension, hypoglycemia, constipation, diarrhea, and vomiting) did not significantly differ between the two groups (*p* > 0.05). However, our assessment of glycemic safety was limited to recorded hypoglycemic events. We did not systematically monitor hyperglycemia, which is a known pharmacological effect of corticosteroids and may have been underreported in the prednisone group. Nevertheless, the incidence of respiratory tract infections was significantly lower in the study group than in the control group (*p* < 0.05) (Table 3).

**Table 3:** Comparison of adverse reactions between the two groups.

Factor	Control group (*n* = 73)	Study group (*n* = 77)	*t*/χ^2^	*p*
Hypotension				
Yes	1	0	0.001	0.979
No	72	77		
Hypoglycemia				
Yes	5	0	3.537	0.06
No	68	77		
Constipation				
Yes	1	0	0.001	0.979
No	72	77		
Diarrhea				
Yes	8	3	2.751	0.097
No	65	74		
Vomiting				
Yes	1	1	0.003	0.953
No	72	76		
Respiratory infection				
Yes	16	7	4.749	0.029
No	57	70		

### Comparison of blood pressure and heart rate parameters between the two groups

Blood pressure and heart rate were monitored during follow-up visits as per institutional protocol. Table 4 shows that no statistically significant differences were found between the two groups in any cardiovascular parameter assessed (all *p* > 0.05). No cases of bradycardia (heart rate < 90 bpm) were documented in either group during the treatment period.

**Table 4:** Comparison of blood pressure and heart rate parameters between the two groups.

Factor	Control group (*n* = 73)	Study group (*n* = 77)	*t*	*p*
Systolic blood pressure after treatment (mmHg)	89.62 ± 17.58	88.75 ± 17.31	0.305	0.761
Diastolic blood pressure after treatment (mmHg)	56.34 ± 11.27	55.81 ± 11.05	0.292	0.771
Heart rate after treatment (bpm)	118.52 ± 10.42	117.06 ± 9.87	0.879	0.381

### Comparison of growth and development between the two groups

After 3–6 months of treatment, infants in the control group had significantly lower height, weight, and head circumference than those in the study group (*p* < 0.05) (Table 5).

**Table 5:** Comparison of growth and development between the two groups of infants.

Factor	Control group (*n* = 73)	Study group (*n* = 77)	*t*/χ^2^	*p*
Body length after treatment	65.25 ± 2.06	71.21 ± 2.57	15.588	<0.001
Body weight after treatment	6.27 ± 0.48	6.94 ± 0.53	8.141	<0.001
Head circumference after treatment	41.13 ± 2.30	44.51 ± 2.12	9.372	<0.001

### Comparison of Gesell developmental scale scores between the two groups

After 3–6 months of treatment, the study group showed significantly higher scores than the control group on all functional domains of the Gesell Developmental Scale, with these domains including gross motor, fine motor, adaptive behavior, language, and personal–social behavior (*p* < 0.05) (Table 6).

**Table 6:** Comparison of Gesell developmental scale scores between the two groups of infants.

Factor	Control group (*n* = 73)	Study group (*n* = 77)	*t*/χ^2^	*p*
Head circumference after treatment	92.50 ± 3.19	95.85 ± 3.30	6.309	<0.001
Fine motor domain score after treatment	91.03 ± 3.32	95.62 ± 3.61	8.091	<0.001
Adaptive behavior domain score after treatment	91.29 ± 3.55	95.65 ± 3.51	7.560	<0.001
Language domain score after treatment	91.51 ± 4.23	94.74 ± 4.58	4.487	<0.001
Personal–social behavior domain score after treatment	91.19 ± 2.04	95.07 ± 2.43	10.558	<0.001

## Discussion

Our study retrospectively analyzed 150 infants with proliferative IHs to compare differences in efficacy, safety, physical growth, and neurodevelopment after 3–6 months of treatment with oral propranolol and those after treatment with oral prednisone. Our results showed that compared with the prednisone group, the propranolol group experienced markedly better clinical efficacy, with overall fewer adverse reactions. Additionally, the propranolol group demonstrated superior outcomes in terms of height, weight, head circumference, and scores across all functional domains of the Gesell Developmental Scale (gross motor, fine motor, language, adaptive behavior, and personal–social behavior) than the control group. These findings provide strong evidence-based support for the use of propranolol as a first-line systemic therapy for proliferative IHs.

In terms of efficacy, our results are consistent with the findings of numerous previous studies (15–17). Propranolol has been reported to be highly effective in reducing proliferative IHs while maintaining a favorable safety profile (18–20). Several clinical cohort studies and retrospective experiences have shown that propranolol achieves high overall improvement rates with rare serious adverse events (21–23). By contrast, traditional corticosteroid treatments, such as prednisone, although effective, are more likely to cause systemic side effects than propranolol (24).

Regarding the impact on growth and development, our study found that after 3–6 months of treatment, infants in the propranolol group had significantly greater height, weight, and head circumference than those in the prednisone group. Additionally, retrospective studies have reported that infants receiving long-term propranolol generally achieve normal developmental milestones and physical growth (14). These findings suggest that compared with corticosteroids, propranolol interferes less with physical development in infants.

Regarding neurodevelopment, assessment using the Gesell Developmental Scale showed that the DQ scores in the propranolol group were higher than those in the prednisone group across multiple domains, including gross motor, fine motor, language, adaptive behavior, and personal–social behavior. This result indicates that during the short-term treatment period (3–6 months), propranolol was not associated with detectable adverse effects on motor or cognitive abilities as measured by the Gesell Scale. Nevertheless, given the short follow-up duration and the use of a single assessment tool in our study, these neurodevelopmental findings should be considered exploratory rather than conclusive. Research on the effects of propranolol on cognitive and behavioral development in infants remains limited. Some studies have reported a slight decline in DQ after long treatment durations (e.g., 6–9 months); however, these studies were based on small sample sizes (14).

Mechanistically, propranolol exerts its therapeutic effects through β-adrenergic receptor blockage, vasoconstriction, endothelial cell proliferation inhibition, and apoptosis promotion (25, 26). In addition, it may suppress HIF-1α and downregulate angiogenic factors, such as VEGF, thereby facilitating the involution of hemangiomas (27). By contrast, while corticosteroids also inhibit proliferation, their systemic hormonal effects are more likely than those of propranolol to interfere with endocrine function, bone metabolism, and energy balance in pediatric patients. This difference may explain the inferior outcomes in growth and developmental assessments observed in the prednisone group.

The observed differences in growth parameters between the two groups are clinically meaningful. Corticosteroids are known to inhibit growth hormone secretion and bone formation, leading to linear growth suppression and delayed weight gain, particularly with prolonged use (28). By contrast, propranolol, which acts primarily through β-adrenergic blockade, does not interfere with the hypothalamic–pituitary–growth axis and is therefore less likely to affect somatic growth than corticosteroids (14). While animal studies have raised concerns about the potential effect of β-blockers on central nervous system maturation, our short-term data did not reveal any detrimental effects on neurodevelopment; instead, infants in the propranolol group showed higher DQ scores than those in corticosteroid group. This finding may be attributable to the better overall clinical status, fewer disease-related complications, or reduced systemic side effects in the propranolol group relative to in the prednisone group (29, 30). However, we acknowledge that our follow-up duration is too short to draw definitive conclusions on long-term neurocognitive outcomes, and the observed differences may also reflect baseline parental or environmental factors not captured in this retrospective analysis.

Despite the significance of our findings, our study has several limitations. First, as a retrospective study, it may be subject to follow-up bias or incomplete adverse event documentation. Second, we only assessed short-term outcomes within 3–6 months of treatment and lacked long-term follow-up data (e.g., at 1 or 2 years). While we excluded infants with known anemia and chronic diseases on the basis of medical records, we did not systematically screen for subclinical anemia or other undiagnosed conditions that might affect growth parameters. Although the proportion of preterm infants was similar between groups, we did not apply separate neurodevelopmental assessment criteria or growth charts for preterm versus term infants. This approach may have influenced the interpretation of developmental outcomes. We did not specifically analyze the distribution of complicated hemangiomas (those with bleeding or ulceration), which could potentially influence treatment response and developmental outcomes, between the two groups. The standard treatment durations differed inherently between the two groups (3–6 months for propranolol versus 4–8 weeks for prednisone), and we did not perform stratified adjustment for treatment length. This limitation may have introduced confounding effects on the comparative evaluation of efficacy and growth outcomes. Glycemic safety was assessed only through documented hypoglycemia events, without the systematic monitoring of hyperglycemia or continuous blood glucose profiling, limiting the completeness of safety comparisons between the two groups, particularly given prednisone’s known hyperglycemic effect. Although blood pressure and heart rate were monitored per institutional protocol, our cardiovascular safety conclusions should be interpreted with caution given our study’s retrospective design and incomplete data capture. Neurodevelopmental outcomes were assessed using only the Gesell Developmental Schedules during a short follow-up period. The sensitivity of this single instrument is limited, and we did not employ complementary tools, such as parent-reported questionnaires or long-term cognitive assessments. Therefore, the observed differences in DQ scores should be interpreted with caution and do not imply long-term neurodevelopmental superiority. In addition, although the Gesell Developmental Schedules were used to evaluate development, the sensitivity of this tool is limited, and complementary assessments, such as parent-reported questionnaires, were not included. Finally, this work is a single-center study, and its sample characteristics and clinical management practices may not be broadly generalizable.

On the basis of the above limitations, future studies should adopt prospective, multicenter designs with large sample sizes and extended follow-up periods to evaluate comprehensively the long-term effects of propranolol on height, weight, head circumference, and cognitive/behavioral development in children. Additionally, refined and multidimensional developmental assessment tools (such as the Bayley Scales of Infant Development and the Wechsler Intelligence Scale for Children), along with parent-reported questionnaires, should be incorporated to enhance assessment sensitivity. Moreover, for patients at risk of rebound growth, further investigation is needed to clarify the relationship among discontinuation strategies, tapering speed, and recurrence rates.

## Conclusion

We suggest that in infants with proliferating hemangiomas, oral propranolol may offer advantages over oral prednisone in terms of therapeutic efficacy, safety, physical growth, and neuropsychological development. Our findings provide additional support for the clinical use of propranolol as a first-line therapy. However, given our study’s limited sample size and short follow-up period, large prospective studies with extended observation periods are needed to confirm our preliminary observations and evaluate long-term safety and optimal treatment strategies further.

## Data Availability

The raw data supporting the conclusions of this article will be made available by the authors, without undue reservation.
